# Comment on “Advancements and Challenges in Tongue Transplantation: A Systematic Review and Meta‐Analysis”

**DOI:** 10.1002/hsr2.72565

**Published:** 2026-05-25

**Authors:** Rajeh Sharef Ruzayqat

**Affiliations:** ^1^ Faculty of Medicine Hebron University Hebron Palestine

## Transparency Statement

The lead/corresponding author, Rajeh Sharef Ruzayqat, affirms that this manuscript is an honest, accurate, and transparent account of the work being reported; that no important aspects of the work have been omitted; and that any discrepancies from the work as planned, and, if relevant, registered, have been explained.

To the Editor,

We read with interest the recent article by Manea et al. (2025), which evaluated tongue transplantation outcomes through a systematic review and meta‐analysis. The authors included six studies (*n* = 51) and presented a forest plot to summarize the findings [1].

According to Table 2 in the original article by Manea et al., the included studies comprise heterogeneous evidence types, including “Birchall et al., with code (T1)” classified as a commentary [2], and “Chi et al., with code (T4)” classified as a review [3] article. While the inclusion of such sources may be appropriate for contextual discussion, their incorporation into the quantitative synthesis (meta‐analysis) raises concerns.

According to Table 2 (column 3) in the original article by Manea et al., both the commentary and the review article do not report a number of cases. Despite this, these studies appear to have been included in the quantitative analyses, including the forest plot presented as (Figure 3) in the original article by Manea et al. The inclusion of studies without clearly reported case numbers or extractable quantitative data raises methodological concerns about how they contributed to the pooled estimates and graphical outputs.

In this context, a descriptive synthesis may have been more appropriate than formal meta‐analysis. Clarification regarding the inclusion criteria for the quantitative analysis and the construction of the forest plot would improve transparency and interpretability of the findings.

## Author Contributions

**Rajeh Sharef Ruzayqat:** writing – review and editing, writing – original draft.

## Funding

The author has nothing to report.

## Ethics Statement

Ethical approval was not required for this article, as it does not involve human participants, animal subjects, or the collection of new primary data.

## Conflicts of Interest

The author declares no conflicts of interest.

## Data Availability

No new data were created or analyzed in this work. The information discussed is derived from previously published articles cited in the manuscript.
